# An Improved Autonomous Emergency Braking Algorithm for AGVs: Enhancing Operational Smoothness Through Multi-Stage Deceleration

**DOI:** 10.3390/s25072041

**Published:** 2025-03-25

**Authors:** Wenbo Li, Junting Qiu

**Affiliations:** 1 School of Mechanical Engineering, University of Shanghai for Science and Technology, Shanghai 200093, China; 2235051120@st.usst.edu.cn; 2 College of Mechanical Engineering, Zhejiang University of Technology, Hangzhou 310014, China

**Keywords:** AGV, AEB algorithm, path planning, obstacle avoidance algorithm, velocity smoothing, industrial automation, SLAM, motion control, emergency stop avoidance

## Abstract

The automated guided vehicle (AGV) is widely used in industrial environments for goods transportation. However, issues such as mechanical wear, reduced battery life, navigation error accumulation, and decreased operational efficiency caused by frequent starts and stops need to be addressed. This paper proposes an improved Autonomous Emergency Braking (AEB) algorithm to tackle these problems. The algorithm employs a stepwise deceleration strategy, effectively reducing the frequency of sudden stops and enhancing the system’s operational smoothness. The AEB algorithm not only considers straight-line driving scenarios but also optimizes deceleration strategies for turning scenarios, adjusting the deceleration detection range according to the turning trajectory. Additionally, a velocity smoothing algorithm is designed to ensure that speed changes during deceleration are gradual, avoiding abrupt speed variations that could impact the system. The feasibility of the AEB algorithm is validated through testing on actual equipment, and its performance is compared to that of a conventional emergency stop strategy. Experimental results show that the AEB algorithm significantly reduces the number of sudden stops, improves the AGV’s operational smoothness and safety, and demonstrates excellent adaptability and robustness across different operational conditions.

## 1. Introduction

AGVs are mobile robots widely used in industry to transport goods from point A to point B. Their core functions include task allocation, path planning, localization, motion planning, and vehicle management [1]. AGVs help manufacturers and operators minimize costs, enhance flexibility, prevent single points of failure, and operate continuously in labor-intensive and accident-free workplaces [2]. Currently, mainstream obstacle avoidance methods primarily rely on Simultaneous Localization and Mapping (SLAM) technology [3], which includes Radar SLAM and Visual SLAM. Radar SLAM constructs maps and performs simultaneous localization by obtaining environmental depth information through laser scanning. Visual SLAM, using cameras as its information source, determines an object’s actual distance and direction based on its position in an image [4]. SLAM is practically applicable in scenarios where predefined maps are challenging, and it has been widely adopted in emerging fields such as augmented reality and autonomous driving [5]. Additionally, SLAM enables mobile robots to perceive their surroundings and determine their position within the environment [6]. In this study, SLAM provides obstacle point cloud data for the AGV’s obstacle avoidance algorithm along its travel path.

Many researchers have focused on AGV obstacle avoidance strategies that primarily involve actively bypassing external obstacles. Daniel Teso-Fz-Betoño et al. [7] implemented an obstacle avoidance function using a “move-to-point” logic, where speed and direction were calculated based on the proportional distance between the target and the AGV. Yong Yuan et al. [8] employed Kalman filtering to estimate the position of dynamic obstacles, constructing a velocity obstacle buffer that allowed the AGV to use the predicted position of obstacles for avoidance. They also developed an objective scoring function that balanced efficiency and safety, selecting the highest-rated speed as the candidate speed for the next moment. Sam Weckx et al. [9] designed a model based on model predictive control that enabled AGVs to avoid obstacles in narrow corridors. However, this approach may lead to unexpected collisions in environments where objects change rapidly. Additionally, AGVs may encounter situations where they cannot surpass obstacles in narrow corridors, necessitating a stop behind the obstacle. This scenario bears similarities to the AEB algorithm [10] already applied in motor vehicles.

The AEB algorithm has proven effective in preventing collisions in both vehicles [11,12] and two-wheeled motor vehicles [13,14]. LU Jiaye et al. [15] designed a three-level control strategy algorithm for the AEB system based on the Time to Collision (TTC). LIU Shijiang et al. [16] studied three models—the TTC model, Mazda model, and Honda model—finding that the TTC model exhibited the best longitudinal collision avoidance performance. Regarding the vehicle itself, the radar detection angle and radar detection range influence collision avoidance [17]. As for the working environment, factors such as the ground slope, friction [18], and weather conditions [19] impact collision avoidance. From these studies, it is evident that AEB in motor vehicles can effectively prevent collisions and reduce accidents by decelerating or even performing an emergency stop.

Some researchers have already studied AGV obstacle avoidance strategies based on the implementation of deceleration zones to reduce speed, a mechanism similar to the AEB algorithm. Ákos Cservenák [20] implemented an emergency stop when an external object comes very close to an AGV. Ladislav Krkoška et al. [21] designed two response zones, one near and one far, at the front and rear of an AGV. When an external object entered the far response zone, the AGV prepared an obstacle avoidance strategy, and when the object entered the near response zone, the AGV executed an emergency stop.

However, frequent emergency braking in AGVs reduces efficiency, accelerates mechanical wear, shortens the battery life, and increases navigation errors, ultimately affecting the overall productivity. One major challenge is that, unlike autonomous vehicles, AGVs have limited computational resources. Autonomous driving AEB systems rely on multi-sensor fusion with LiDAR, millimeter-wave radar, and cameras, supported by high computational power. In contrast, AGVs typically use only LiDAR, process far less data, and cannot support complex deep learning or large-scale data fusion algorithms.

To address these limitations, we propose an improved AEB algorithm optimized for AGVs. Instead of detecting all obstacles in the surrounding environment, our algorithm focuses solely on obstacles within the AGV’s future path. By predicting potential collisions and adjusting speed through progressive deceleration, it ensures smoother operation while minimizing emergency stops. This targeted detection approach significantly reduces computational demands and shortens the processing time. Emergency braking is only triggered when an obstacle is too close and avoidance is infeasible. The design enables efficient obstacle avoidance even in low-computation environments, particularly in indoor, flat terrains where path alteration is often constrained.

The main contributions of this paper are as follows:

1. An AEB algorithm was designed that enables an AGV to decelerate step by step when an external obstacle is detected ahead, with an emergency stop triggered only when necessary. This minimizes emergency stops and mitigates issues caused by abrupt halts. Additionally, the algorithm is optimized for low-computation AGVs by detecting only obstacles in their future path, reducing the processing time and computational demands.
2. In addition to considering the case where the AGV travels in a straight line, the AEB algorithm also accounts for turning scenarios, simulating the turning trajectory and adjusting the deceleration detection range based on the distance to obstacles along the trajectory.
3. Building upon the step-by-step deceleration mechanism of the AEB algorithm, an advanced speed smoothing algorithm was designed to ensure that the AGV’s speed transitions gradually rather than abruptly between discrete deceleration levels. This approach mitigates the impact on the system caused by sudden speed changes during stepwise deceleration.
4. The AEB algorithm was tested on actual equipment, demonstrating its feasibility. Parameters were adjusted and experiments were conducted to compare the performance advantages of the AEB algorithm with conventional emergency stop obstacle avoidance.

## 2. AEB Algorithm

### 2.1. Kinematic Modeling of the AGV Chassis

In order to simplify calculations, the AGV is represented by a single point that reflects its position and orientation in the world coordinate system, which is used to determine the AGV’s state. Before studying the AEB algorithm for AGVs, it is necessary to clarify the conversion process from the AGV’s equivalent point motion state to the actual controllable wheel parameters. This involves the modeling and analysis of forward and inverse kinematics. The process requires the development of a kinematic model based on the structural characteristics of the AGV, such as the wheel layout and drive method. Using the velocity and directional information of the equivalent point, control parameters such as the rotational speed and steering angle of the drive wheels are derived. This foundational research provides theoretical support for the real-time performance and accuracy of the AEB algorithm and ensures that the algorithm can efficiently and stably execute braking operations under complex operating conditions.

This study involves two typical AGV chassis configurations. The first is a differential-drive chassis, where the equivalent point is located at the center of the AGV. This configuration is characterized by strong symmetry and relatively simple kinematic modeling, making it widely used in industrial automation due to its ease of control and straightforward trajectory planning. The second is a single-steering wheel chassis, where the equivalent point is typically offset from the center of the AGV due to factors such as the cargo load or structural design. This offset affects the vehicle’s motion characteristics, introducing additional complexity into trajectory prediction and motion control. Despite this complexity, the single-steering wheel chassis is commonly used in AGV applications, particularly in forklifts and other logistics vehicles, due to its maneuverability and adaptability to varying load conditions. Understanding its kinematic modeling is essential in accurately predicting motion constraints and ensuring precise control in automated operations.

**Figure 1:**
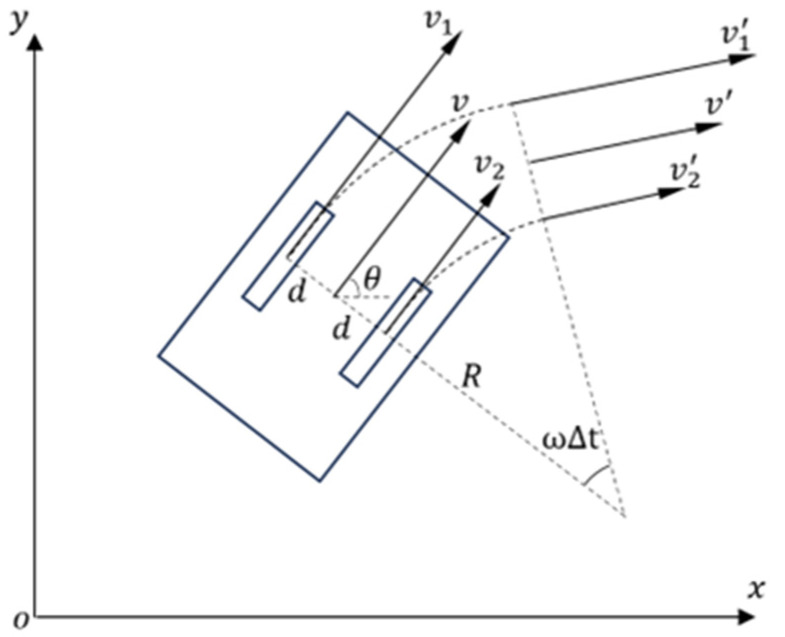
Kinematic modeling of the differential drive chassis AGV.

The kinematic modeling of the differential drive chassis AGV is shown in Figure 1.

The differential drive chassis AGV is driven by two symmetrically arranged differential wheels along the center of the AGV body, achieving directional changes through velocity differences. The kinematic modeling for a differential drive chassis AGV is as follows:

Let the velocity of the equivalent point be v. The velocities of the two drive wheels are denoted as $v_{1}$ and $v_{2}$, respectively. The distance between the two drive wheels is 2d, and the distance from the equivalent point to the center of rotation is R.

The kinematic forward and inverse solution formulas are as follows:

(1) $$v_{1}=\frac{R+2d}{R+d}v\;v_{2}=\frac{R}{R+d}v$$

During the motion of the differential drive chassis AGV, let the time step be ∆t, and denote the coordinates of the equivalent point as $P=\left(x,y\right)$ with an orientation angle θ relative to the positive *x*-axis of the world coordinate system. The state update equations for the equivalent point are as follows:

(2) $$\left\{\begin{array}{c} x\left(t+∆t\right)=x\left(t\right)+v\cos\theta ∆t\\ y\left(t+∆t\right)=y\left(t\right)+v\sin\theta ∆t\\ \theta \left(t+∆t\right)=\theta \left(t\right)+\omega ∆t \end{array}\right.$$

**Figure 2:**
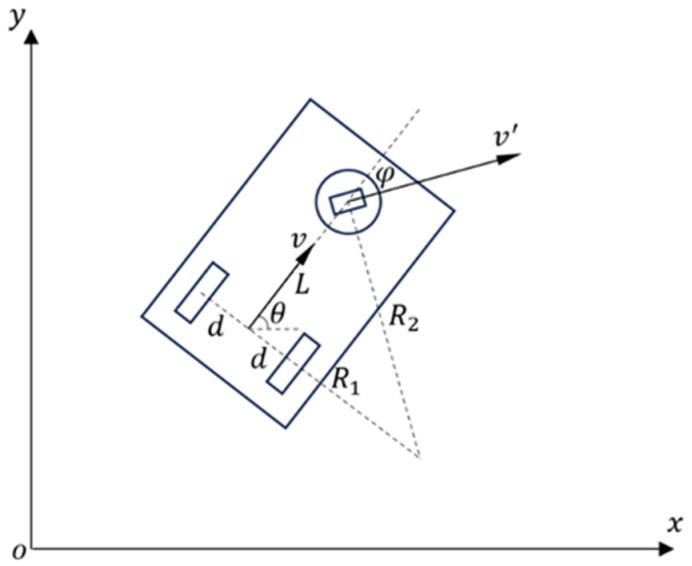
Kinematic modeling of the single-steering wheel chassis AGV.

The kinematic modeling of the single-steering wheel chassis AGV is shown in Figure 2.

The single-steering wheel chassis AGV is driven by a steering wheel capable of adjusting both its direction and speed. The kinematic modeling process for a single-steering wheel chassis AGV is as follows:

Let the velocity of the equivalent point be v and the angular velocity be ω. The linear velocity of the steering wheel is denoted as $v^{\prime }$. Define the distance from the equivalent point to the steering wheel center as L. Let φ be the angle between the movement direction of the steering wheel and the equivalent point.

The kinematic forward solution formulas are as follows:

(3) $$v=\frac{sin\left(\varphi \right)}{tan\left(\varphi \right)}v^{\prime }\;\omega =\frac{sin\left(\varphi \right)}{L}v^{\prime }$$

The inverse solution formulas are as follows:

(4) $$v^{\prime }=\sqrt{L^{2}\omega^{2}+v^{2}}\;\varphi ={tan}^{-1}\left(\frac{L\omega }{v}\right)$$

During the motion of the single-steering wheel chassis AGV, let the time step be ∆t, and denote the coordinates of the equivalent point as $P=\left(x,y\right)$ with an orientation angle θ relative to the positive *x*-axis of the world coordinate system. The state update equations for the equivalent point are identical to those of the differential drive chassis AGV and are given as Equation (2).

### 2.2. Introduction to the Principles of the AEB Algorithm

**Figure 3:**
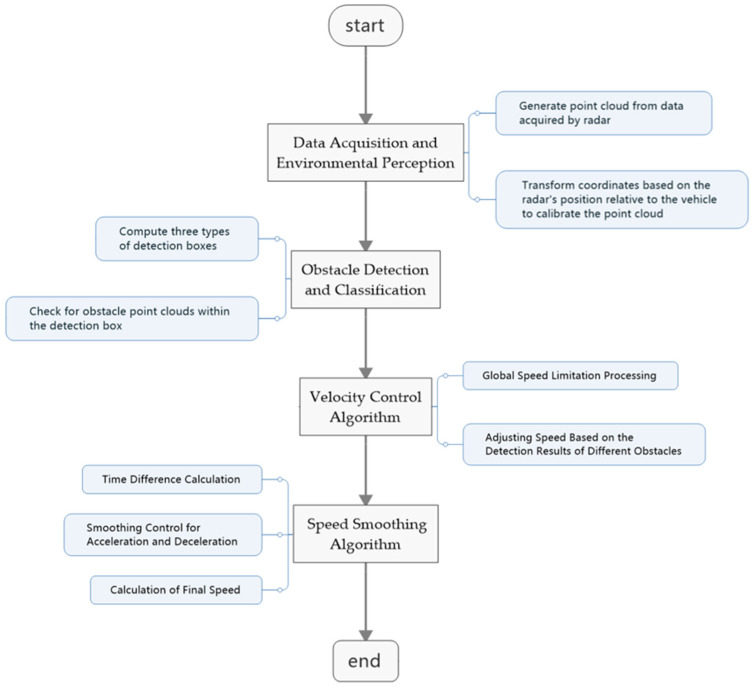
AEB algorithm flowchart.

The AEB algorithm is a technology that enables dynamic obstacle avoidance and safety braking based on real-time sensor data. Its core objective is to assess potential collision threats and implement appropriate braking measures, even in scenarios where navigation and localization are ineffective or restricted. As shown in Figure 3, the application of the AEB algorithm in AGVs consists of the following key modules:

#### 2.2.1. Data Acquisition and Environmental Perception

The AEB module receives point cloud data from sensors (message type: sensor_msgs::PointCloud2). Typically, the reference coordinate frame of the point cloud is the sensor’s local coordinate frame, sensor_frame. To enable these data to be used for global decision-making, they must be transformed into the AGV’s base coordinate frame, base_link_frame. This transformation can be formally described as follows:

(5) $$p_{base}=T_{sensor-to-base}\cdot p_{sensor}$$

where $p_{base}$ and $p_{sensor}$ represent the coordinates of the point cloud in the base coordinate frame and the sensor coordinate frame, respectively:

(6) $$p_{sensor}=\left[\begin{array}{c} x_{sensor}\\ y_{sensor}\\ z_{sensor}\\ 1 \end{array}\right],\;p_{base}=\left[\begin{array}{c} x_{base}\\ y_{base}\\ z_{base}\\ 1 \end{array}\right]$$

$T_{sensor-to-base}$ is the transformation matrix representing the pose conversion from the sensor coordinate frame to the base coordinate frame. Its specific values are determined based on the installed position of the LiDAR sensor:

(7) $$T_{sensor-to-base}=\left[\begin{array}{cccc} r_{11} & r_{12} & r_{13} & p_{x}\\ r_{21} & r_{22} & r_{23} & p_{y}\\ r_{31} & r_{32} & r_{33} & p_{z}\\ 0 & 0 & 0 & 1 \end{array}\right]$$

where $R=\left[\begin{array}{ccc} r_{11} & r_{12} & r_{13}\\ r_{21} & r_{22} & r_{23}\\ r_{31} & r_{32} & r_{33} \end{array}\right]$ is the rotation matrix and $t=\left[\begin{array}{c} t_{x}\\ t_{y}\\ t_{z} \end{array}\right]$ is the translation vector.

#### 2.2.2. Obstacle Detection and Classification

To effectively detect obstacles and implement appropriate avoidance measures, the AEB algorithm first utilizes point cloud data acquired from sensors such as LiDAR and depth cameras to construct a series of rectangular detection boxes. These detection boxes are used to predict the AGV’s future positions over different time steps and serve as the basis for subsequent obstacle avoidance decisions. By accurately computing these detection boxes, the algorithm can simulate the potential relative position changes between the AGV and obstacles in the environment during motion.

The detection region’s bounding box is classified into three state types: EMERGENCY_STOP, SPEED_STOP, and DECELERATION, with the priority decreasing in the same order. EMERGENCY_STOP indicates that the obstacle is too close, requiring the AGV to stop immediately; SPEED_STOP indicates that the obstacle is relatively close, prompting the AGV to gradually reduce its speed until it comes to a halt; and DECELERATION indicates that the obstacle is at a safe distance, allowing the AGV to decelerate according to the set parameters. When the obstacle’s point cloud enters the corresponding detection box, the system will adopt the appropriate obstacle avoidance strategy based on the current state.

The generation of the detection box follows the steps below:

1. Determine the vertices of the AGV’s outline.

The AGV chassis is approximated as a rectangle, with its vertices represented by two-dimensional coordinates $p_{i}=\left(x_{i},y_{i}\right)$. The bounding box is generated based on the physical outline of the AGV (i.e., the planar area occupied by the AGV). The outline vertices are typically defined with the AGV’s center as the origin. The formula is as follows:

(8) $$footprint=\left[\left(x_{1}\right.,\left.y_{1}\right),\left(x_{2}\right.,\left.y_{2}\right),\left(x_{3}\right.,\left.y_{3}\right),\left(x_{4}\right.,\left.y_{4}\right)\right]$$

In the code, these vertices are passed as parameters and stored in a list.

2. Calculate the detection box.

The AEB algorithm generates a bounding box for obstacle detection and avoidance decision-making using real-time velocity information from sensors and predefined parameters. The shape and size of the bounding box are determined by the AGV’s physical outline and its current pose, with the four vertex coordinates calculated based on the AGV’s physical dimensions and current position. The algorithm continuously updates these bounding boxes to reflect the AGV’s motion trajectory and the surrounding environment in real-time, enabling proactive collision risk assessment and response.

- Generation of the Emergency Stop Bounding Box.

The emergency stop bounding box (corresponding to the EMERGENCY_STOP state detection box) is a rectangle defined by four vertices with the AGV’s center as the origin. The dimensions of this rectangle are slightly larger than the AGV’s physical dimensions. The generation formula is as follows:

(9) $$emergency\_stop\_footprint=\left[\left(x_{1}\right.,\left.y_{1}\right),\left(x_{2}\right.,\left.y_{2}\right),\left(x_{3}\right.,\left.y_{3}\right),\left(x_{4}\right.,\left.y_{4}\right)\right]$$

In the code, these vertices are passed as parameters and stored in a list.

- Generation of the Speed Stop Bounding Box.

The speed stop bounding box (corresponding to the SPEED_STOP state detection box) is primarily generated based on the linear velocity feedback from the AGV’s sensors. The algorithm predicts the positions and quaternions (collectively referred to as the pose) of a series of future time points in the global coordinate system, starting from the AGV’s current coordinate system, based on the existing linear velocity. Then, using the coordinates of the AGV’s outline vertices in the local AGV coordinate system and the previously calculated pose, the algorithm computes the coordinates of the bounding box vertices corresponding to the AGV’s position at future time points in the current AGV coordinate system. With these vertex coordinates, the corresponding rectangle is then determined. The detailed steps are as follows:

First, calculate the minimum stopping distance $s_{predict\_path\_len}$ required for the AGV to come to a stop from its current linear velocity. The formula is as follows:

(10) $$s_{predict\_path\_len}=\frac{v_{f}^{2}}{2d_{max}}$$

where $v_{f}$ is the real-time linear velocity feedback from the AGV’s motor encoder, calculated as the square root of the sum of squares of the linear velocities in the x and y directions, $v_{fx}$ and $v_{fy}$. $d_{max}$ is the maximum deceleration set for the AGV. For safety reasons, $d_{max}$ is generally smaller than the AGV’s maximum achievable deceleration. The calculation of $s_{predict\_path\_len}$ is used to define the range of the speed stop bounding box. If an obstacle enters these boxes, then the AGV must immediately stop with maximum deceleration to ensure safety.

Next, the number of detection boxes N is calculated. The formula is as follows:

(11) $$N=\frac{s_{predict\_path\_len}}{S_{dis\_spacing}}$$

where $S_{dis\_spacing}$ is the distance interval between the centers of adjacent detection boxes, which is set as a parameter before the program runs. The result of the formula may yield a remainder. If a remainder exists, then the result is incremented by 1, and the distance between the centers of the last and second-to-last boxes is adjusted to equal the remainder. The calculation of N is used to determine the total number of detection boxes, which will be used for stepwise iterative counting in the subsequent process.

After calculating N, the algorithm will compute the pose of all detection boxes, with the calculation method being as follows:

First, we calculate the small time difference ∆t between adjacent detection boxes, using the following formula:

(12) $$∆t=\frac{S_{dis\_spacing}}{v_{f}}$$

Next, a loop is initiated to iterate over the calculated values, with the number of iterations equal to N.

The algorithm calculates the angular displacement ∆θ of the AGV within the time interval ∆t, based on the angular velocity ω of the current AGV coordinate axes:

(13) ∆θ=ω∆t

where ω is the real-time angular velocity feedback from the AGV’s motor encoder.

The encoder feedback velocities $v_{fx}$ and $v_{fy}$ are transformed based on the rotation matrix of the respective coordinate system. The calculation formula is as follows:

(14) $$\left[\begin{array}{c} v_{x}^{\prime }\\ v_{y}^{\prime } \end{array}\right]=\left[\begin{array}{cc} cos\theta_{pose} & -sin\theta_{pose}\\ sin\theta_{pose} & cos\theta_{pose} \end{array}\right]\cdot \left[\begin{array}{c} v_{x}\\ v_{y} \end{array}\right]$$

$\left[\begin{array}{c} v_{x}\\ v_{y} \end{array}\right]$ represents the linear velocity vector in the detection box coordinate system (assuming that the AGV’s linear velocity remains constant).

$\left[\begin{array}{cc} cos\theta_{pose} & -sin\theta_{pose}\\ sin\theta_{pose} & cos\theta_{pose} \end{array}\right]$ represents the transformation between the detection box coordinate system and the AGV’s current coordinate system.

$\theta_{pose}$ is the rotation angle corresponding to each detection box, initially set to θ and incremented by ∆θ in each iteration of the loop.

$\left[\begin{array}{c} v_{x}^{\prime }\\ v_{y}^{\prime } \end{array}\right]$ represents the linear velocity vector in the AGV’s current coordinate system, transformed from the detection box coordinate system.

Next, the position of the detection box’s coordinate system origin is calculated based on the obtained velocity, using the following formula:

(15) $$\left\{\begin{array}{c} p_{x}=v_{x}^{\prime }∆t\\ p_{y}=v_{y}^{\prime }∆t \end{array}\right.$$

Based on $\theta_{pose}$, the angular orientation of the detection box coordinate system is obtained, and it is represented in quaternion form along with the previously calculated vector $\left[\begin{array}{c} p_{x}\\ p_{y} \end{array}\right]$ to fully represent the pose of the detection box coordinate system. After N iterations, the final pose data are stored in an array containing multiple pose objects, where each pose object represents the position and orientation of the AGV at a specific moment.

After calculating the pose of the detection box coordinate system, the algorithm calculates and publishes the coordinates of each detection box’s rectangular vertices in the AGV’s current coordinate system.

The algorithm first retrieves the four vertices of the detection box in the detection box coordinate system by reading the AGV’s outline vertices (footprint), with the vertices represented as follows:

(16) $$\left\{\begin{array}{c} p_{1}=\left(x_{1},y_{1}\right)\\ p_{2}=\left(x_{2},y_{2}\right)\\ p_{3}=\left(x_{3},y_{3}\right)\\ p_{4}=\left(x_{4},y_{4}\right) \end{array}\right.$$

Then, the pose transformation matrix is used to convert the representation of these four vertices in the detection box coordinate system into their representation in the AGV’s current coordinate system. The formula is as follows:

(17) $$\left[\begin{array}{c} x_{i}^{\prime }\\ y_{i}^{\prime }\\ 0\\ 1 \end{array}\right]=\left[\begin{array}{cccc} cos\theta_{pose} & -sin\theta_{pose} & 0 & p_{x}\\ sin\theta_{pose} & cos\theta_{pose} & 0 & p_{y}\\ 0 & 0 & 1 & 0\\ 0 & 0 & 0 & 1 \end{array}\right]\cdot \left[\begin{array}{c} x_{i}\\ y_{i}\\ 0\\ 1 \end{array}\right]$$

$\left[\begin{array}{c} x_{i}\\ y_{i}\\ 0\\ 1 \end{array}\right]$ represents the vertices of the detection box in the detection box coordinate system.

$\left[\begin{array}{cccc} cos\theta_{pose} & -sin\theta_{pose} & 0 & p_{x}\\ sin\theta_{pose} & cos\theta_{pose} & 0 & p_{y}\\ 0 & 0 & 1 & 0\\ 0 & 0 & 0 & 1 \end{array}\right]$ represents the pose transformation between the detection box coordinate system and the AGV’s current coordinate system.

$\theta_{pose}$ is the rotation angle corresponding to each detection box.

$p_{x}$ and $p_{y}$ are the coordinates of the detection box’s coordinate system origin in the AGV’s current coordinate system.

$\left[\begin{array}{c} x_{i}^{\prime }\\ y_{i}^{\prime }\\ 0\\ 1 \end{array}\right]$ represents the linear velocity vector in the detection box coordinate system, expressed in the AGV’s current coordinate system.

i is the index of the vertex coordinates, which can take any value from the set {1,2,3,4}.

After the four sets of coordinates are calculated, they are stored in the array RectPoints[i] and then published.

- Generation of the Deceleration Bounding Box.

Simultaneously to the generation of the speed stop bounding boxes, the deceleration bounding box (corresponding to the DECELERATION state detection box) is also generated following the same set of rules. After generating these deceleration bounding boxes, the algorithm will generate additional deceleration bounding boxes according to the same rules. The formula for calculating the number of additional deceleration bounding boxes is as follows:

(18) $$N^{\prime }=max\left(\frac{S_{detect}}{S_{dis\_spacing}}-N,N\right)$$

$S_{detect}$ is the additional detection distance added to the previous speed stop bounding boxes, and it is set as a parameter before the program runs. Since the data undergo iteration during the calculation of pose transformation for the bounding boxes, the data used to generate the additional deceleration bounding boxes are based on the speed stop bounding boxes. Therefore, $\frac{S_{detect}}{S_{dis\_spacing}}$ must subtract the previously generated number of bounding boxes, N.

This formula generates rectangular boxes within the specified detection range, ensuring that the minimum number of bounding boxes generated is N.

3. Determining Whether an Obstacle is Inside the Bounding Box.

Obstacle detection is performed by analyzing the geometric relationship between the point cloud data and the detection box. The core algorithm used is the ray-casting method. In this method, a ray is cast from the point $P_{obs}$ in any arbitrary direction, and the number of intersections between the ray and the boundaries of the rectangular bounding box is calculated. If the ray intersects the boundary of the bounding box an odd number of times, then the point is considered to be inside the bounding box; if the number of intersections is even, then the point is considered to be outside the bounding box.

The algorithm casts a horizontal ray (i.e., along the *x*-axis direction) from the obstacle point $P_{obs}\left(x_{obs},y_{obs}\right)$ to the right. To determine whether the point is inside the bounding box, we need to check whether the ray intersects with any of the four edges of the rectangular bounding box. Each edge of the bounding box can be represented as a line segment, defined as follows:

(19) $$\left\{\begin{array}{c} Edge1:\;P1(x_{1},y_{1})\;P2(x_{2},y_{2})\\ Edge2:\;P2(x_{2},y_{2})\;P3(x_{3},y_{3})\\ Edge3:\;P3(x_{3},y_{3})\;P4(x_{4},y_{4})\\ Edge4:\;P4(x_{4},y_{4})\;P1(x_{1},y_{1}) \end{array}\right.$$

The algorithm first iterates through the four edges, denoting the coordinates of the two points on each edge as $P_{start}\left(x_{start},y_{start}\right)$ and $P_{end}\left(x_{end},y_{end}\right)$. If $y_{start}=y_{end}$, then it is assumed that there is no intersection between the obstacle point and this edge. If $y_{obs}<min\left(y_{start},y_{end}\right)$ or $y_{obs}>max\left(y_{start},y_{end}\right)$, then it is also assumed that there is no intersection between the obstacle point and this edge. When neither of the above two conditions hold, the x-coordinate of the intersection point between the edge and the ray can be calculated using the following formula:

(20) $$x_{intersect}=\frac{\left(y_{obs}\right.\left.-y_{start}\right)\left(x_{end}-x_{start}\right)}{y_{end}-y_{start}}+x_{start}$$

where $x_{intersect}$ is the x-coordinate of the intersection point. If $x_{intersect}>x_{obs}$, then it is determined that there is an intersection.

#### 2.2.3. Velocity Control Algorithm

1. Global Speed Limitation Processing;

First, the velocity feedback from the AGV encoder will be processed to ensure that it does not exceed the system’s maximum speed limit. Let the feedback velocity command be represented as $twist=\left(v_{x},\;v_{y},\;\omega \right)$, where $v_{x}$ is the linear velocity of the AGV in the *x*-axis direction, $v_{y}$ is the linear velocity in the *y*-axis direction, and ω is the angular velocity during AGV turning. The algorithm will process the input velocity based on the maximum speed limit $V_{limit}$ to obtain the processed velocity command:

(21) $$\left\{\begin{array}{c} v_{xlimited}=min\left(v_{x},V_{xlimit}\right)\\ v_{ylimited}=min\left(v_{y},V_{ylimit}\right)\\ \omega_{limited}=min\left(\omega ,\Omega_{limit}\right) \end{array}\right.$$

where $V_{xlimit}$, $V_{ylimit}$, and $\Omega_{limit}$ are the system-defined maximum linear velocities along the *x*-axis and *y*-axis components and the maximum angular velocity, respectively.

2. Adjusting Speed Based on the Detection Results of Different Obstacles.

Since the velocity used in the following calculations is a composite of the components in the x and y directions, a conversion is required. The steps are as follows:

Using the Pythagorean theorem, the original linear velocities in the x and y directions are combined, as expressed by the following equation:

(22) $$v_{limited}=\sqrt{v_{xlimited}^{2}+v_{ylimited}^{2}}$$

where $v_{x}$ and $v_{y}$ are the linear velocity components of the AGV in the *x*-axis and *y*-axis directions, respectively.

- Emergency Stop.

When an obstacle is detected in close proximity to the AGV, the system will immediately set the AGV’s speed to zero:

(23) $$v_{x,final}=0,\;\;v_{y,final}=0,\;\;\omega_{final}=0$$

At this point, both the AGV’s linear velocity and angular velocity will be set to zero, and the AGV will come to an immediate stop. Meanwhile, the system will call set_holding_status to set the AEB status to emergency stop: aeb_type::EMERGENCY_STOP. The current AEB status will then be published.

- Speed Stop.

When an obstacle is detected and a collision with the AGV’s future position is imminent, requiring the AGV to stop, the system will perform a smooth deceleration based on the current speed and the distance between the AGV and the obstacle, ensuring collision avoidance while maintaining smooth motion. For safety reasons, after the speed reaches zero, regardless of any changes in the obstacle’s status, the algorithm will maintain this state for a few seconds, with the duration adjustable. After this delay, the algorithm will call set_holding_status and set it to the normal stop state: aeb_type::SPEED_STOP.

- Deceleration.

When the system detects an obstacle and deceleration is required, the AGV’s speed will be adjusted based on the distance to the obstacle and the current speed. The AGV will decelerate to the minimum safe speed. If the distance to the obstacle is $d_{obstacle}$, then the AGV’s final speed $v_{final}$ can be calculated using the following formula:

(24) $$v_{final}=min\left(v_{limited},f\left(d_{obstacle}\right)\right)$$

Here, $f\left(S_{obstacle}\right)$ is a function used to adjust the target speed based on the distance to the obstacle. It is generally configured according to the specific requirements of the application. The core of the function consists of two lists (unit: meters).

(25) $$aeb\_obstacle\_distance=\left[d_{1},d_{2},d_{3},d_{4},d_{5}\right]\;aeb\_obstacle\_speed=\left[v_{1},v_{2},v_{3},v_{4},v_{5}\right]$$

The values in the two lists correspond to each other. When the distance between the AGV’s center and the specified predicted bounding box of the obstacle is less than the value of aeb_obstacle_distance, the maximum speed will be reduced to the corresponding value in aeb_obstacle_speed. The values and the number of elements in the lists can be flexibly adjusted based on actual requirements. However, based on safety considerations and practical use, the following constraints apply:

The obstacle distance $d_{n}$ and the speed $v_{n}$ must be in increasing order, and the speed corresponding to the obstacle must be less than the limit speed. The calculation formula for the limit speed is as follows:

(26) $$v_{max}=\sqrt{2ad}$$

Here, a is the maximum deceleration that the AGV can achieve.

This limitation prevents the AGV from traveling too fast to safely stop within the corresponding distance, ensuring the safety of the algorithm. Additionally, for safety reasons, after the AGV has stopped, regardless of any changes in the obstacle’s status, the algorithm will maintain this state for several seconds, with the specific duration adjustable. After this delay, the algorithm will update the AEB status to the deceleration state, aeb_type::DECELERATION, and publish this status.

#### 2.2.4. Speed Smoothing Algorithm

When adjusting the speed, the system can introduce smoothing control to avoid abrupt changes in velocity, which could cause significant inertial impacts or vibrations, thereby affecting the stability and safety of the AGV. The principle of the speed smoothing algorithm is as follows:

1. Time Difference Calculation.

Calculate the time interval Δt between the current time $t_{cur}$ and the last smoothing control time $t_{last\_smooth}$, as expressed by the following formula:

(27) $$\Delta t=min\left(t_{cur}-t_{last\_smooth},0.1\right)$$

The 0.1 s interval is used to ensure that the smoothing control update frequency is not lower than 10 Hz. If there is a delay time, then it can be added to Δt.

2. Smoothing Control for Acceleration and Deceleration.

When the target speed is greater than the feedback speed, the AGV needs to accelerate. During acceleration, the speed sent to the AGV is constrained by the AGV’s maximum acceleration, and the acceleration rate cannot exceed the maximum allowable acceleration. The maximum acceleration speed $v_{cmd\_limit}$ sent to the AGV is as follows:

(28) $$v_{cmd\_limit}=v_{f}+a∆t$$

where $v_{f}$ is the current feedback linear velocity and a is the AGV’s maximum acceleration.

Then, the actual speed $v_{cmd\_final}$ sent to the AGV is limited to the minimum value between the final speed $v_{final}$ calculated by the deceleration algorithm and the maximum allowable speed $v_{cmd\_limit}$:

(29) $$v_{cmd\_final}=min\left(v_{final},v_{cmd\_limit}\right)$$

This operation further limits the acceleration rate after the global speed limitation, preventing excessive acceleration.

When the target speed is less than the feedback speed, the AGV needs to decelerate. During deceleration, the speed sent to the AGV is constrained by the AGV’s maximum deceleration, and the deceleration rate cannot exceed the maximum allowable deceleration. The maximum deceleration speed $v_{cmd\_limit}$ sent to the AGV is as follows:

(30) $$v_{cmd\_limit}=v_{f}-d∆t$$

where d is the AGV’s maximum deceleration.

Then, the actual speed $v_{cmd\_final}$ sent to the AGV is limited to the maximum value between the final speed $v_{final}$ calculated by the deceleration algorithm and the maximum allowable deceleration speed $v_{final}$:

(31) $$v_{cmd\_final}=max\left(v_{final},v_{cmd\_limit}\right)$$

3. Calculation of Final Speed.

Based on the smoothed control speed $v_{cmd\_final}$, update the original velocity components on the *x*-axis and *y*-axis:

(32) $$rate=\frac{v_{cmd\_final}}{v_{f}}\;v_{cmd\_final\_x}=rate\cdot v_{fx}\;v_{cmd\_final\_y}=rate\cdot v_{fy}$$

#### 2.2.5. State Management and Publishing

The AEB algorithm leverages the topic mechanism of the ROS (Robot Operating System) to efficiently publish key information to various modules of the AGV, enabling functions such as emergency braking, speed adjustment, and state transmission. This design ensures that the AGV system can quickly respond to potential threats in complex environments while maintaining a highly scalable modular structure.

The processed velocity information is published through the topic /control_cmd_vel. This topic contains the linear and angular velocity commands generated by the AEB algorithm based on real-time environmental analysis. After subscribing to this topic, the motion control module converts these commands into specific actions for the AGV, such as deceleration, acceleration, or emergency stop. This mechanism ensures that the AEB algorithm can rapidly respond to dynamic changes in obstacles, thus ensuring the safe operation of the AGV.

Additionally, the obstacle detection status and related parameter information are published through the topic /aeb/status. This topic includes real-time detection results for obstacles, such as their type, distance, and relative position, and the current operational status of the AEB system (e.g., emergency stop, deceleration, or normal operation). These data provide support for system monitoring and analysis and also offer an interface for remote operation and data logging modules.

Through the collaborative operation of these two features, the AEB algorithm efficiently transmits speed adjustment and environmental perception information, enhancing the system’s response capability and operational reliability under complex conditions. The ROS-based design also ensures the system’s excellent scalability, providing convenient conditions for future functional optimization and algorithm upgrades.

## 3. Experimental Testing and Analysis

To comprehensively validate the effectiveness and robustness of the proposed AEB algorithm under various operating conditions, in this study, we designed a series of targeted experimental scenarios for both quantitative and qualitative analysis. Through these experiments, we aimed to simulate typical operational environments and complex situations that an AGV may encounter in actual industrial applications, covering scenarios such as static obstacle handling and obstacle avoidance optimization during path turns. Through these experiments, the performance of the AEB algorithm under different task requirements and environmental variations can be assessed.

**Figure 4:**
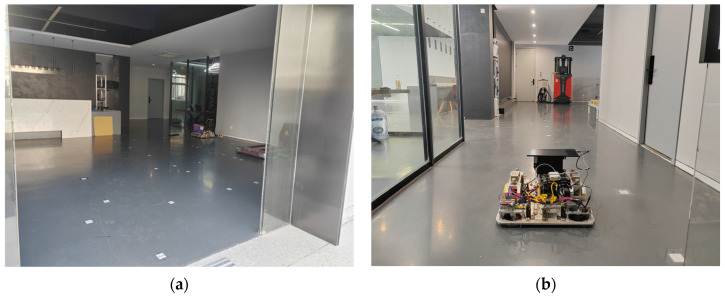
Schematic of the experimental setup: (**a**) 8 m × 6 m area; (**b**) 10 m × 2 m area.

The experimental setup comprises a flat indoor environment, consisting of an 8 m × 6 m rectangular area and a 10 m × 2 m rectangular area to ensure sufficient operational space for testing the AGV’s performance in straight-line driving and turning, as shown in Figure 4.

**Figure 5:**
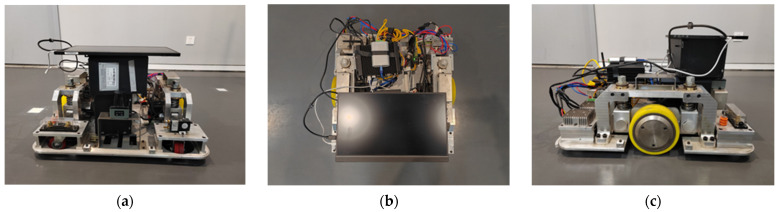

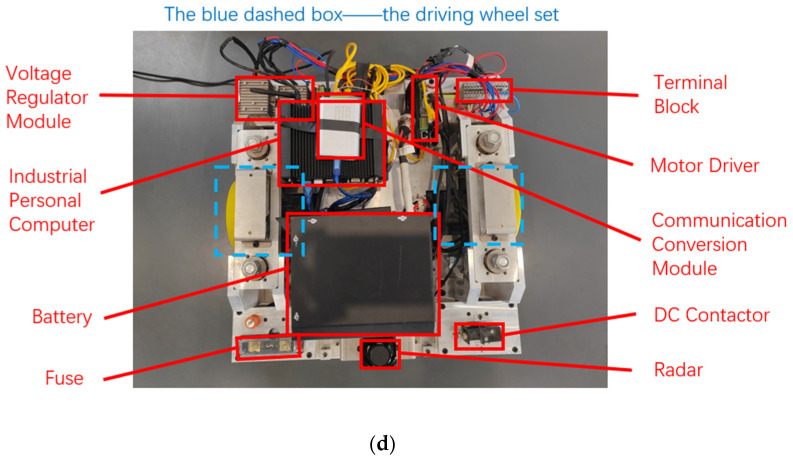
Schematic of the experimental AGV. (**a**) Front view; (**b**) top view; (**c**) left view; (**d**) component diagram.

The experimental equipment consists of an AGV with a differential drive chassis. The AGV is driven by two symmetrically distributed drive wheels, with passive wheels mounted at the four corners of the vehicle to assist in movement. Radar is installed at the front of the vehicle to detect obstacle information. The AGV design meets the typical requirements for industrial application scenarios, with its appearance and component specifications (excluding the external display) shown in Figure 5.

The parameter information for the AGV is shown in Table 1.

**Figure 6:**
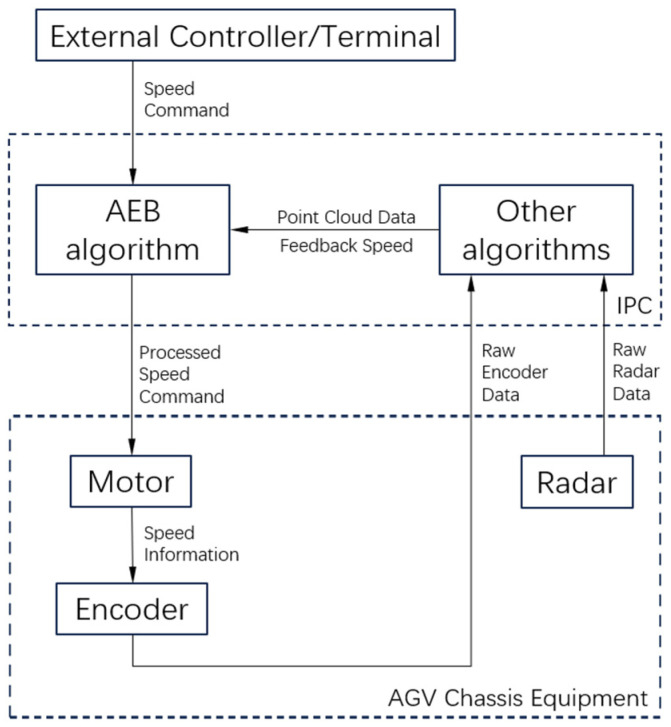
Schematic diagram of the information transmission architecture of the AEB algorithm for the experimental AGV.

The information transmission architecture of the AGV and AEB is shown in Figure 6.

The experimental design takes into account the challenges posed by different operating conditions to the AGV’s navigation and obstacle avoidance capabilities, focusing on the following three aspects:

1. Static Obstacle Detection and Acceleration/Deceleration Experiment.

In this experiment, obstacles are placed at fixed positions. By varying the acceleration and deceleration values, the data obtained are used to analyze the reliability and performance of the AEB algorithm while also optimizing the parameter settings.

2. Turning Obstacle Avoidance Experiment.

This test simulates potential scenarios where the AGV may encounter obstacles during a path turn, assessing the AEB algorithm’s trajectory prediction capability and its accuracy in recognizing and responding to surrounding obstacles.

3. Experiment on Dynamic Obstacle Avoidance at Intersections.

This experiment simulates a warehouse intersection scenario where both an AGV and a moving pedestrian pass through the intersection simultaneously. The goal is to evaluate the AEB algorithm’s ability to prevent collisions and minimize sudden stops when encountering dynamic obstacles.

4. Comparison with the TEB Dynamic Obstacle Avoidance Algorithm.

This experiment compares the TEB and AEB algorithms in a straight-line scenario with a dynamic obstacle appearing at a fixed position for a set duration. The AGV starts from rest, accelerates to a predefined speed, and navigates the obstacle. Key metrics include obstacle avoidance success, emergency stop avoidance, and travel time. TEB’s path deviation is disabled to focus on collision avoidance. The results evaluate motion smoothness and efficiency in constrained environments.

5. Comparison with Conventional Emergency Stop Strategy.

This comparison evaluates the emergency stop performance of the AGV using the AEB algorithm versus the traditional emergency stop strategy under different obstacle response times. By adjusting the speed, acceleration, and obstacle distance, the emergency stop time and avoidance rate are calculated, highlighting the advantages of the AEB algorithm in preventing sudden stops.

### 3.1. Static Obstacle Detection and Acceleration/Deceleration Experiment

This experiment aims to validate the effectiveness of the AEB algorithm during the AGV deceleration process, ensuring that it can achieve smooth and gradual deceleration under varying acceleration and deceleration conditions. By adjusting acceleration and deceleration parameters, the experiment analyzes the variation in the number of deceleration stages and explores the impact of speed step optimization on the deceleration performance.

The AGV is placed in a 10 m × 2 m rectangular field, starting from an initial stationary state, accelerating to a maximum speed of 1.0 m/s and maintaining a constant velocity. A stationary obstacle is placed within the detection area to trigger the AEB algorithm. By recording the number of emergency stops during the gradual deceleration process, the effectiveness of the avoidance strategy in reducing emergency stops is assessed. Different deceleration values are set to calculate the actual acceleration or deceleration during the process, verifying that the AEB algorithm can adapt well to different conditions based on the parameter settings. Additionally, the generation of speed–time graphs provides an important reference for optimizing multi-stage deceleration parameters.

Due to field constraints and safety considerations, the experimental baseline parameters are shown in Table 2. The experiment was conducted based on the baseline parameters, with adjustments made to both acceleration and deceleration to study whether changes in parameters could effectively alter the output speed of the AEB algorithm and improve the smoothness of the speed transition.

**Figure 7:**
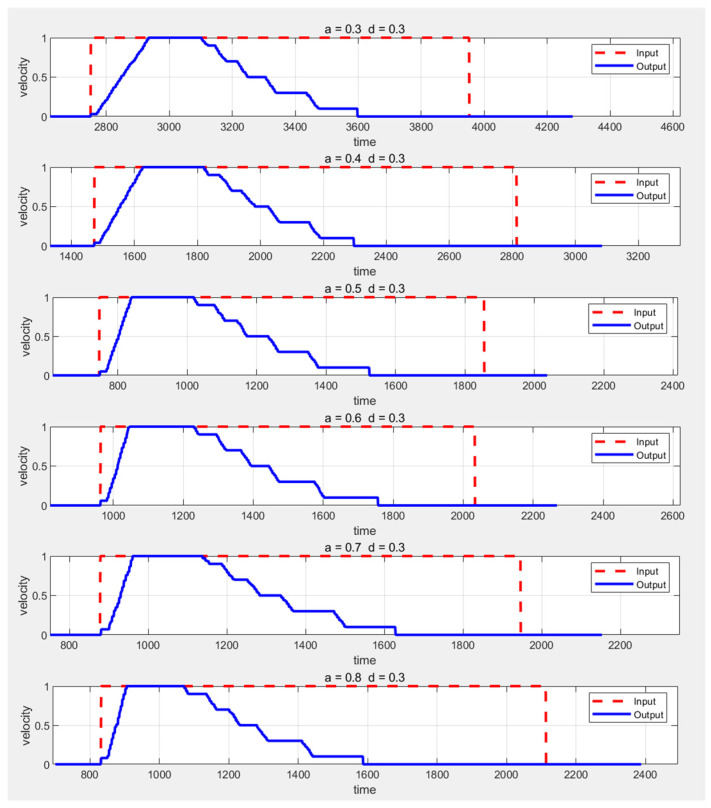
Comparison of AEB input and output speeds when acceleration changes.

The experiment first keeps the deceleration constant and then varies the acceleration values from 0.3 m/s^2^ to 0.8 m/s^2^, observing the experimental outcomes. The AEB input-output graph is shown in Figure 7. In the figure, the red dashed line represents the commanded speed input to the AEB algorithm, while the blue solid line represents the speed sent to the AGV chassis after processing by the AEB.

**Figure 8:**
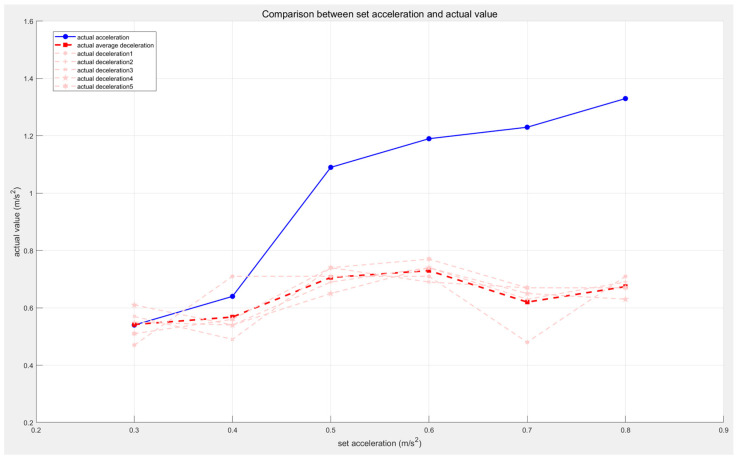
Comparison of acceleration and deceleration when acceleration changes.

From these six speed–time plots, it can be observed that after receiving the command speed, the AGV begins accelerating at a constant rate until it reaches the maximum speed of 1 m/s, maintaining this speed for a period of time. When an obstacle is detected, the AGV starts decelerating in stages, with each stage’s speed consistently matching the set value, demonstrating a smooth deceleration process. The final segment shows a deceleration from 0.1 m/s to 0, with its slope approaching infinity, confirming the successful activation of the emergency stop function in the AEB algorithm. By comparing the set acceleration and deceleration values with the actual values, the effectiveness of the algorithm’s parameter adjustment can be verified. The actual acceleration is calculated by determining the rate of change in speed from 0 to 1 m/s, while the actual deceleration is calculated by analyzing the start and end times of each deceleration phase and averaging all deceleration stages. Specific values for acceleration and deceleration are detailed in Table 3 and Figure 8.

As shown in Figure 8, the actual acceleration is positively correlated with the set acceleration, while the actual deceleration remains around 0.6 m/s^2^. This result indicates that the AEB algorithm can adapt to AGVs with different accelerations.

**Figure 9:**
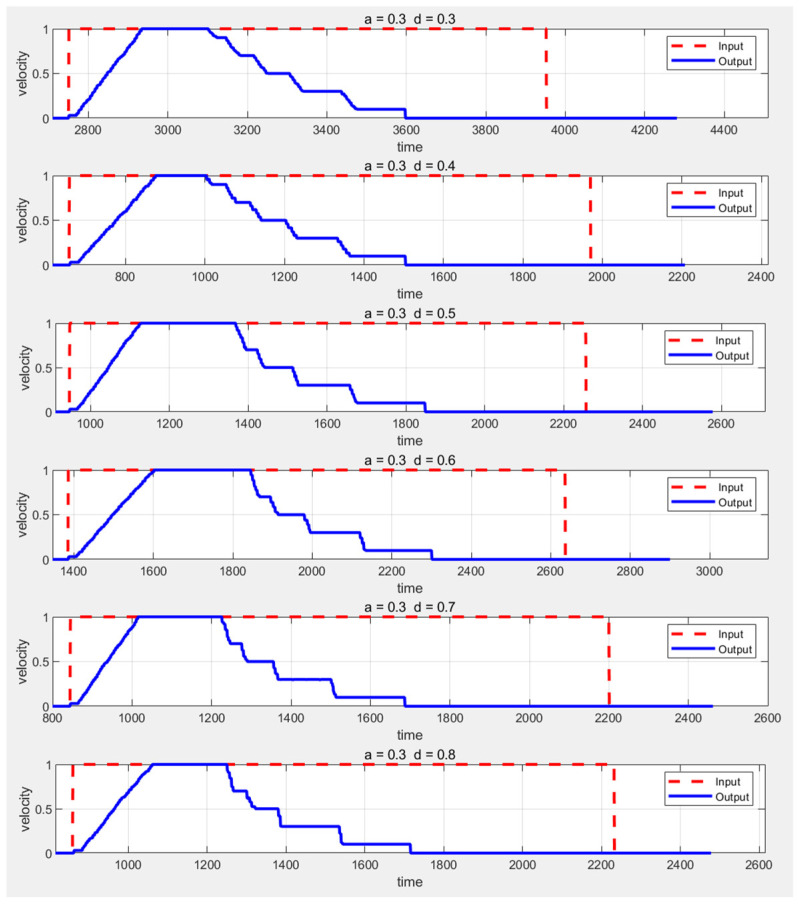
Comparison of AEB input and output speeds when deceleration changes.

Next, the acceleration setpoint was kept constant, and the deceleration was varied from 0.3 m/s^2^ to 0.8 m/s^2^ to observe the experimental effect. The AEB input–output graphs are shown in Figure 9.

**Figure 10:**
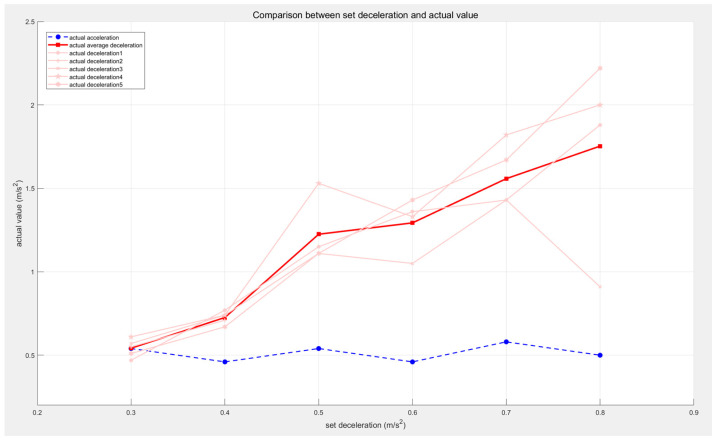
Line chart of acceleration and deceleration values when the deceleration is changed.

The specific values of the acceleration and deceleration set for velocity smoothing and the final output acceleration and deceleration are provided in Table 4 and Figure 10.

As shown in Figure 10, the actual deceleration is positively correlated with the set deceleration, while the actual acceleration remains at 0.5 m/s^2^. This result indicates that the AEB algorithm can adapt to AGVs with varying deceleration requirements. Unlike in the previous experiment, the number of deceleration stages begins to decrease when the acceleration is set to 0.5 m/s^2^. This is because the next multi-stage deceleration condition is triggered before the speed reaches 0.9 m/s. To optimize the multi-stage deceleration effect, the step size between adjacent speed levels can be reduced in higher speed ranges, achieving a smoother and more gradual deceleration process.

In both experiments, the AGV successfully implemented multi-stage deceleration based on the principles of the AEB algorithm, and no emergency stop occurred except when approaching the obstacle. By adjusting acceleration and deceleration separately, the experiments validated the theory that the AEB algorithm could accommodate AGVs with different acceleration and deceleration characteristics. The phenomenon of a decrease in the number of deceleration stages led to the conclusion that in higher speed ranges, the optimization of the deceleration effect can be achieved by reducing the step size between adjacent speed levels. The experiments also revealed that although the set acceleration and deceleration values were positively correlated with the actual values, there was still some discrepancy between them. This discrepancy may be due to hardware performance limitations or errors in the calculation of acceleration and deceleration. Future research should further investigate the causes of this discrepancy to improve the system’s accuracy and adaptability.

### 3.2. Turning Obstacle Avoidance Experiment

This experiment aims to verify the effectiveness of the AEB algorithm in the obstacle avoidance process of an AGV, ensuring that it can achieve smooth, staged deceleration under different turning radii and velocity conditions, thus avoiding emergency stop phenomena. By adjusting the angular velocity to change the turning radius, the impact of the gradient obstacle avoidance strategy on system stability is analyzed and the reliability of the AGV’s obstacle avoidance during turning is validated. The experiment also focuses on the adaptability of the AEB algorithm in complex paths, providing a basis for optimizing the obstacle avoidance strategy and enhancing system safety.

**Figure 11:**
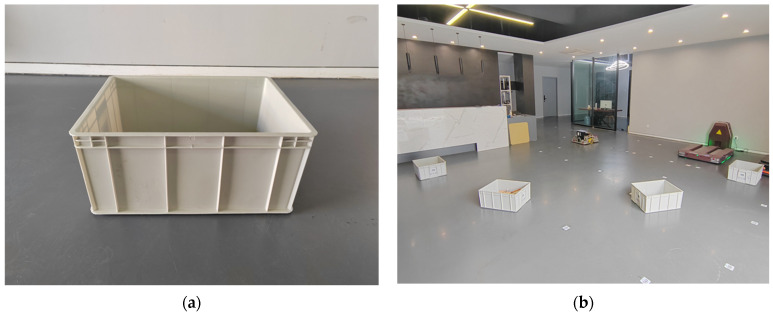
Experimental obstacles and obstacle setup. (**a**) Obstacles; (**b**) obstacle setup.

The AGV starts from an initial stationary state, maintaining an angular velocity $\omega_{z}=\omega$, then accelerates to a linear velocity of $v_{x}=0.7\;m/s$ and continues to move at a constant speed. A set of stationary obstacles is placed in the front region to trigger the AEB algorithm. The AGV’s trajectory, as well as the acceleration and deceleration segments, are recorded to assess the effectiveness of its obstacle avoidance strategy during turns. The obstacles are rectangular boxes with the dimensions 0.54 m × 0.42 m × 0.24 m. They are evenly distributed within a circle of radius 4 m in the first quadrant, centered at the AGV’s starting point, with the AGV’s forward direction along the positive *x*-axis and its left side along the positive *y*-axis. The actual arrangement of the obstacles is shown in Figure 11.

By setting different angular velocities, the turning radius of the AGV is adjusted to verify that the AEB algorithm can achieve the expected gradient-based obstacle avoidance effect at different turning radii. The parameter settings are shown in Table 2 and are consistent with the baseline parameters of Experiment 3.1.

**Figure 12:**
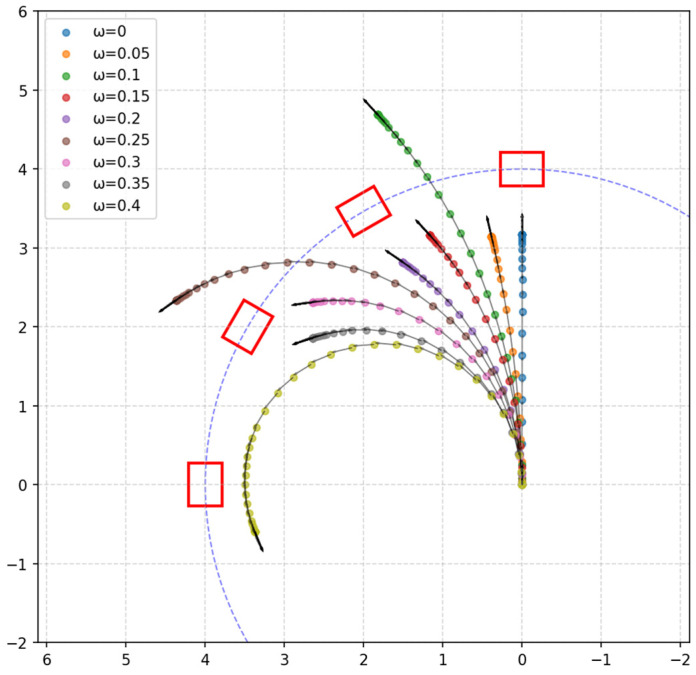
Trajectory plot of the AGV under different angular velocities.

Figure 12 shows the motion trajectories of the AGV under different angular velocity conditions, where the red box represents the obstacle group and the colored scatter points represent the real-time trajectory of the AGV, with equal time intervals between adjacent points. By observing the changes in spacing between the scatter points, the variations in the AGV’s speed during motion can be effectively analyzed. Further examination of the trajectory reveals that as the angular velocity changes, the AGV is able to adjust its path in real time based on the AEB algorithm, successfully avoiding collisions with obstacles during the turning process and thereby ensuring the effective implementation of the obstacle avoidance strategy. Overall, the AGV can flexibly adjust its motion strategy at different angular velocities, maintaining safe operation and achieving the expected obstacle avoidance function in complex environments. This demonstrates that the AEB algorithm not only ensures the safe operation of the AGV under conventional conditions but also exhibits the capability to adapt to different turning radii and motion environments, showcasing high stability and reliability.

**Figure 13:**
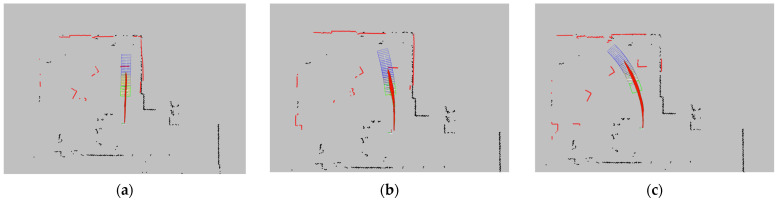

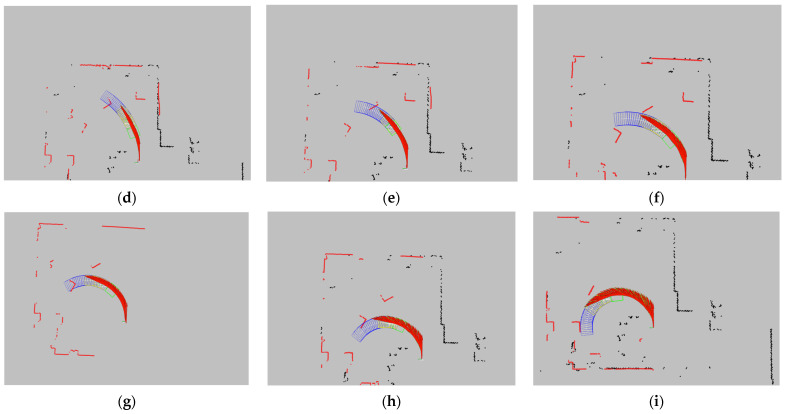
Deceleration zone and speed stop zone during the experiment. The red points represent real-time LiDAR scan data, while the black points correspond to stored map point cloud data, which is not directly relevant to this experiment. (**a**) ω=0; (**b**) ω=0.05; (**c**) ω=0.1; (**d**) ω=0.15; (**e**) ω=0.2; (**f**) ω=0.25; (**g**) ω=0.3; (**h**) ω=0.35; (**i**) ω=0.4.

Figure 13 illustrates the deceleration zones (blue) and speed stop zones (yellow) during the experiment, showing their positions along the AGV’s trajectory. When an obstacle enters one of these zones, the corresponding deceleration is triggered, as observed in Figure 13a,b,d,e,g,h. This result indicates that the setup of these zones effectively supports the deceleration process, enabling the AGV to smoothly adjust its speed and avoid collisions. These zones allow the AGV to respond promptly and take appropriate actions when obstacles enter the defined areas, ensuring safe operation in complex environments.

In the experiments, the AGV successfully implemented multi-stage deceleration based on the AEB algorithm principles, avoiding emergency stops in all trials and ensuring stable system operation. Notably, as the AGV approached obstacles, it gradually decelerated to optimize the obstacle avoidance process, effectively preventing abrupt stops and maintaining continuity and stability in the avoidance strategy. By adjusting different angular velocities to alter the turning radius, the experiment confirmed that the AEB algorithm could still achieve the desired gradient obstacle avoidance effect at varying turning radii, with the AGV effectively avoiding obstacles during turns, thereby ensuring safe operation.

### 3.3. Experiment on Dynamic Obstacle Avoidance at Intersections

**Figure 14:**
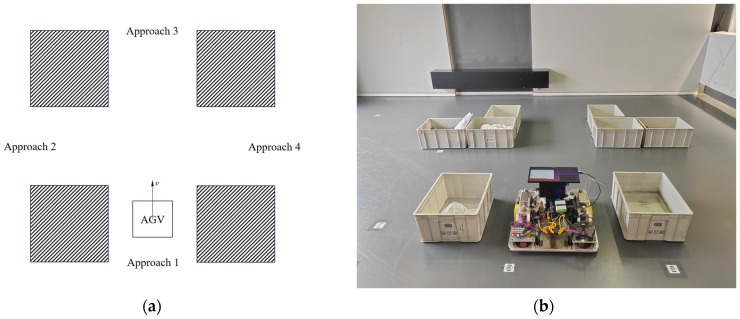
The schematic diagram of the intersection and the actual placement of obstacles. (**a**) Schematic diagram of the intersection. (**b**) Actual placement of obstacles.

This experiment recreates a warehouse intersection environment where an AGV and a moving pedestrian traverse the intersection at the same time. The objective is to assess the effectiveness of the AEB algorithm in avoiding collisions and reducing unnecessary emergency stops when dealing with dynamic obstacles. The schematic diagram of the intersection and the actual placement of obstacles are shown in Figure 14.

In this setup, the AGV and the pedestrian start from different entry points of the intersection. The intersection is labeled as follows: the bottom approach is marked as 1, with the remaining approaches labeled sequentially in a clockwise direction as 2, 3, and 4. The AGV’s starting and destination approaches are set as 1 to 2, 1 to 3, and 1 to 4, while the pedestrian’s starting and destination approaches are set as 2 to 3, 2 to 4, and 3 to 4. Each scenario is tested five times, and the avoidance success rate and emergency stop prevention rate are calculated for each case. The parameter settings for the AGV are shown in Table 5.

**Figure 15:**
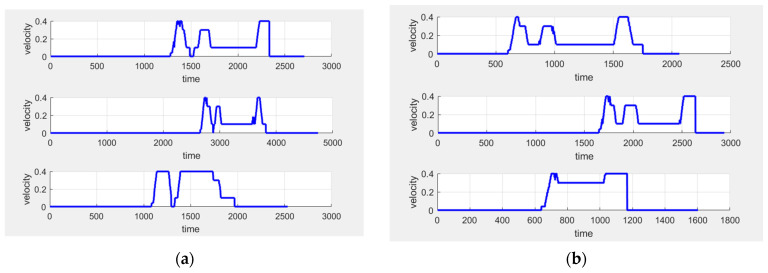
Examples of cases with and without emergency stops. (**a**) Cases with emergency stops; (**b**) cases without emergency stops.

After conducting 45 sets of dynamic obstacle avoidance experiments, the AEB algorithm successfully ensured that the AGV avoided collisions in all cases. When a moving pedestrian was present ahead, the AGV was able to decelerate according to the speed gradient and return to its original set speed once the obstacle moved away. In this experiment, whether an emergency stop was triggered was determined by analyzing the relationship between the output speed of the AEB algorithm and time. Specifically, if the curve reached zero, then an emergency stop was considered to have occurred. Examples of cases with and without emergency stops are shown in Figure 15.

The emergency stop avoidance rate under different conditions is shown in Table 6.

From the table, it can be observed that the AEB algorithm achieves a relatively high emergency stop avoidance rate in most cases. However, in the cases corresponding to the third row, third column and to the fourth row, third column, the emergency stop avoidance rate is relatively low. This indicates that the AEB algorithm has a weaker handling capability for dynamic obstacles obstructing the AGV in the lateral and rear directions. Overall, the experiment demonstrates that the AEB algorithm can effectively prevent emergency stops in most scenarios. In future research, the algorithm can be further optimized to address specific cases with lower emergency stop avoidance rates.

### 3.4. Comparison with the Timed-Elastic-Band (TEB) Dynamic Obstacle Avoidance Algorithm

The TEB algorithm is an optimization-based local path planning method commonly used for mobile robot obstacle avoidance and autonomous navigation. The core idea is to represent the robot’s trajectory as a series of state sequences with time constraints and optimize these states to ensure that the trajectory meets kinematic constraints while dynamically avoiding obstacles.

The purpose of comparing the TEB and AEB algorithms is to evaluate their performance in obstacle avoidance and motion smoothness for AGVs. TEB, as an optimization-based local path planning method, dynamically adjusts the robot’s trajectory to avoid obstacles while maintaining smooth motion. In contrast, the improved AEB algorithm focuses on stepwise deceleration to minimize abrupt stops and enhance stability. This experiment primarily targets narrow corridor scenarios where bypassing obstacles is not feasible. Therefore, in the parameter settings for the TEB algorithm, path deviation is disabled, and only its collision avoidance functionality is utilized. By comparing these two approaches in this study, we aimed to determine their effectiveness in handling dynamic obstacles, ensuring smooth operation, and improving overall navigation efficiency. The key parameter settings are shown in Table 7.

The total length of this test is 4 m, following a straight-line path. The AGV starts from rest and accelerates to a predetermined speed. A dynamic obstacle is placed 3 m ahead of the AGV, which remains in place for 4 s after the AGV starts moving before being removed. The experiment evaluates the AGV’s obstacle avoidance success rate, emergency stop avoidance rate, and travel time from the starting point to the endpoint, providing a comprehensive assessment of the practical performance of the AEB and TEB algorithms.

**Figure 16:**
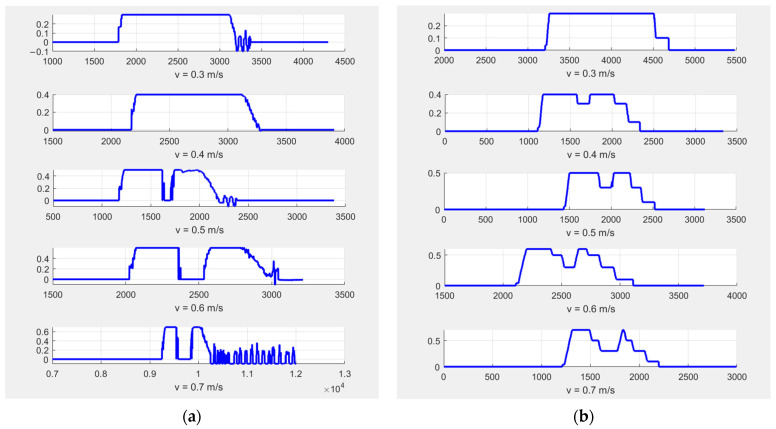
Speed–time curves for the comparison experiment for TEB and AEB algorithms. (**a**) Speed–time curve for the TEB algorithm; (**b**) speed–time curve for the AEB algorithm.

After completing 10 sets of experiments, the results indicate that both algorithms can effectively avoid obstacles and are capable of restarting or accelerating after the obstacle is removed, ultimately reaching the endpoint successfully. Figure 16 presents the speed–time curves for these 10 experiments.

From the images, it can be observed that the TEB algorithm triggers an emergency stop when encountering an obstacle in the path that cannot be bypassed, occurring at speeds of 0.5 m/s, 0.6 m/s, and 0.7 m/s. In contrast, the AEB algorithm, which decelerates in advance, does not trigger an emergency stop under the same conditions. Additionally, the TEB algorithm exhibits unexplained speed oscillations after reaching the target point, which results in small back-and-forth movements in real-world operation. The AEB algorithm, however, does not exhibit this issue. Overall, the AEB algorithm provides smoother motion compared to the TEB algorithm.

We also recorded the travel time from the starting point to the endpoint for both algorithms, as shown in the Table 8.

As shown in the table, at lower set speeds, the TEB algorithm achieves a shorter travel time. However, at higher set speeds, the AEB algorithm benefits from its stepwise deceleration strategy, effectively avoiding emergency stops, which gradually enhances its advantage and results in a shorter time to reach the endpoint.

In conclusion, this experiment demonstrates that when obstacle avoidance through path deviation is not considered, the AEB algorithm provides smoother motion compared to the TEB algorithm. Additionally, AEB effectively prevents emergency stops through a stepwise deceleration strategy, allowing the AGV to maintain a more stable and continuous movement when encountering dynamic obstacles. Furthermore, the experimental results indicate that AEB improves overall operational efficiency by successfully avoiding emergency stops, enabling the AGV to reach the target more quickly. Therefore, in narrow environments or scenarios where bypassing obstacles is not feasible, the AEB algorithm proves to be more advantageous than the TEB algorithm.

### 3.5. Comparison with Conventional Emergency Stop Strategy

This experiment aims to investigate the emergency stop avoidance effect of the AGV when applying the AEB algorithm during multi-stage deceleration under different obstacle reaction times. Specifically, the experiment alters the distance between the obstacle and the AGV’s initial position, setting different speeds and accelerations, and calculates the time at which an emergency stop occurs during the deceleration process. The emergency stop occurrence time is compared between the AEB algorithm and the traditional emergency stop strategy. The primary metrics in the experiment are the emergency stop occurrence time and the Emergency Stop Avoidance Rate ($R_{ESA}$). By varying the reaction times, the experiment analyzes the advantages of the AEB algorithm in avoiding emergency stops.

**Figure 17:**
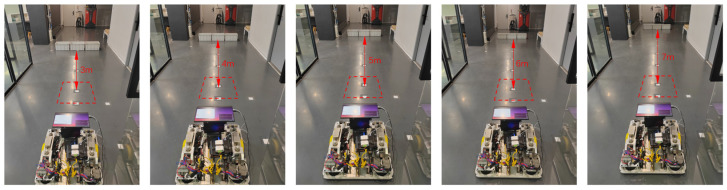
Schematic diagram of obstacle placement at different distances and the AGV’s starting point in the experiment. The red dotted boxes indicate the AGV’s starting point.

The AGV is placed in a 10 m × 2 m rectangular area, with markers spaced 1 m apart, and obstacles and markers are aligned along the center, as shown in Figure 17.

The AGV starts from an initial stationary state, accelerates to its maximum speed, and maintains a constant velocity. Timing begins after passing the starting point and ends when the AGV comes to a stop. In this experiment, the AEB algorithm is configured with single-stage deceleration parameters to simulate the standard emergency stop. The emergency stop time $t_{AEB\_stop}$ using the AEB algorithm’s multi-stage deceleration and the emergency stop time $t_{normal\_stop}$ using conventional emergency braking are measured, allowing for the calculation of the Emergency Stop Avoidance Rate ($R_{ESA}$).

The experiment varies the distance between the obstacle and the starting point, the maximum speed sent to the AGV, and the deceleration rate for the AGV’s multi-stage deceleration. The motion time of the AGV is measured under different conditions. The parameters for both obstacle avoidance strategies and the values of experimental variables are listed in Table 9.

**Figure 18:**
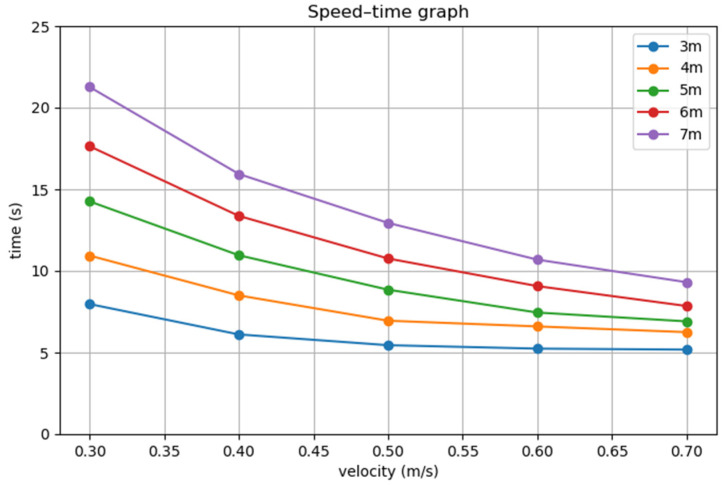
Two-dimensional plot of speed versus emergency stop occurrence time for obstacles at different distances.

After conducting multiple sets of emergency stop experiments using the AEB algorithm with multi-stage deceleration, the corresponding three-dimensional plot of the experimental data is shown in Figure 18.

As shown in Figure 18, there is a significant negative correlation between speed and the emergency stop occurrence time, while the obstacle distance shows a significant positive correlation with the emergency stop occurrence time. This indicates that the experimental data trends align with the theoretical expectations of the AEB algorithm, thus providing a basis for subsequent calculations and analysis.

**Figure 19:**
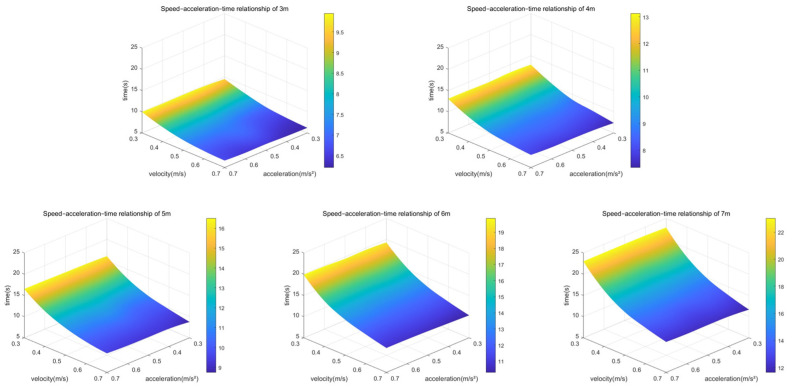
Three-dimensional plot of speed, acceleration, and emergency stop occurrence time corresponding to obstacles at different distances.

After conducting multiple sets of emergency stop experiments using the multi-stage deceleration of the AEB algorithm, the corresponding three-dimensional plot of the experimental data is shown in Figure 19.

As shown in Figure 19, there is a significant negative correlation between speed and emergency stop occurrence time; acceleration and emergency stop occurrence time show a positive correlation, but the relationship is relatively weak; and the distance to the obstacle exhibits a clear positive correlation with the emergency stop occurrence time. These results indicate that the experimental data trends align with the theoretical expectations of the AEB algorithm, thus allowing for subsequent calculations and analyses based on these data.

The emergency stop avoidance rate is derived from the previously calculated emergency stop occurrence time: First, we assume that the obstacle reaction time is $t_{r}$ and the emergency stop occurrence time is t. If $t_{r}<t$, then the obstacle will move away before the emergency stop and the AGV will not perform an emergency stop. If $t_{r}>t$, then the obstacle will not move away before the emergency stop and the AGV will perform an emergency stop. With a fixed obstacle reaction time $t_{r}$, the emergency stop rate using the normal emergency stop deceleration and the emergency stop rate using the AEB algorithm’s multi-level deceleration are given by the following formulas:

(33) $$R_{normal\_stop}=\frac{N\left(t_{r}>t_{normal\_stop}\right)}{N_{normal\_stop}}\;R_{AEB\_stop}=\frac{N\left(t_{r}>t_{AEB\_stop}\right)}{N_{AEB\_stop}}$$

where $N\left(t_{r}>t_{normal\_stop}\right)$ is the number of instances where $t_{r}>t_{normal\_stop}$, and $N_{normal\_stop}$ is the total number of emergency stop experiments using the normal emergency stop deceleration; $N\left(t_{r}>t_{AEB\_stop}\right)$ is the number of instances where $t_{r}>t_{AEB\_stop}$, and $N_{AEB\_stop}$ is the total number of emergency stop experiments using the AEB algorithm’s multi-level deceleration.

After calculating the emergency stop rate for both stop strategies, the emergency stop avoidance rate $R_{ESA}$ for the AEB algorithm is calculated using the following formula:

(34) $$R_{ESA}=\frac{R_{normal\_stop}-R_{AEB\_stop}}{R_{normal\_stop}}$$

Next, by varying $t_{r}$, the emergency stop avoidance rate $R_{ESA}$ for different values of $t_{r}$ can be obtained.

**Figure 20:**
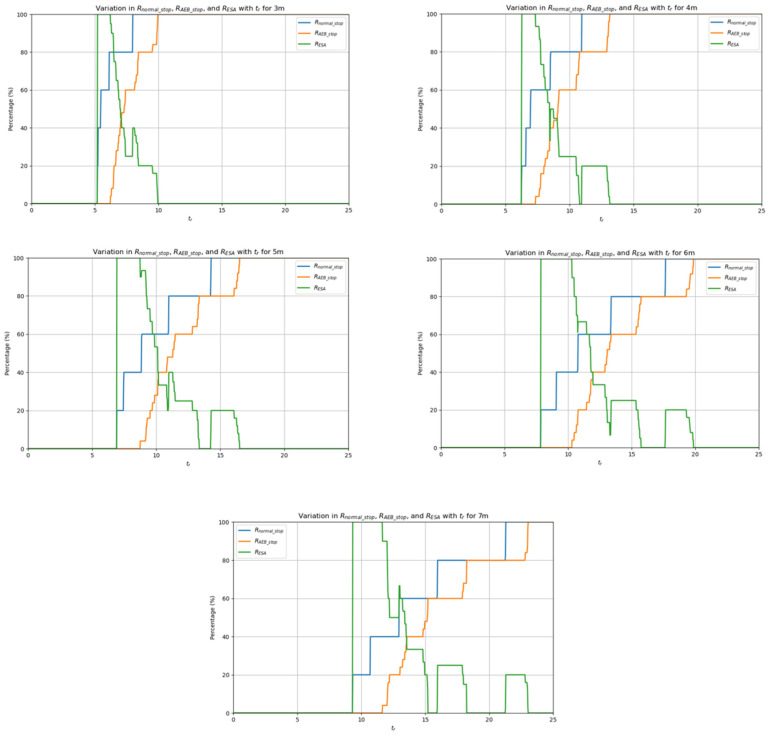
The images of $N_{normal\_stop}$, $N_{AEB\_stop}$, and $R_{ESA}$ as a function of $t_{r}$ for different obstacle distances.

Finally, the images showing $N_{normal\_stop}$, $N_{AEB\_stop}$, and $R_{ESA}$ as a function of $t_{r}$ for different obstacle distances are presented in Figure 20.

Based on the experimental results, the blue curve in Figure 20 represents the relationship between $N_{normal\_stop}$ and time $t_{r}$, the orange curve represents the relationship between AEB algorithm deceleration $N_{AEB\_stop}$ and time $t_{r}$, and the green curve shows how the Emergency Stop Avoidance Rate ($R_{ESA}$) changes with $t_{r}$. From the figure, it can be observed that before $t_{r}$ approaches the normal emergency stop trigger time, neither the AEB algorithm deceleration nor the normal emergency stop deceleration causes an emergency stop, and $R_{ESA}$ is 0. As $t_{r}$ approaches the critical point of normal emergency stop triggering, the AEB algorithm effectively avoids the emergency stop by decelerating in advance, and $R_{ESA}$ reaches 100%. As $t_{r}$ increases, both the AEB algorithm’s and the normal emergency stop’s emergency stop rates start to rise and $R_{ESA}$ gradually decreases. Finally, when both emergency stop rates reach 100%, $R_{ESA}$ drops to 0. After calculating the average value of $R_{ESA}$ over the interval from the first point where $R_{ESA}$ is non-zero to the last point where $R_{ESA}$ is non-zero, the result is shown in Table 10.

The experimental results clearly demonstrate the significant advantages of the AEB algorithm over conventional emergency braking (normal stop). Specifically, the AEB algorithm initiates multi-stage deceleration in advance within the obstacle reaction time, effectively preventing the occurrence of an emergency stop, particularly within specific reaction time ranges. In contrast, conventional emergency braking only begins when the triggering conditions are met, resulting in a higher emergency stop rate and more abrupt responses. By employing the AEB algorithm, the AGV achieves a smoother, more gradual deceleration process, avoiding the discomfort and safety risks associated with sudden stops.

Overall, the AEB algorithm reduced the average emergency stop rate by 42.526% compared to traditional emergency braking, enhancing the AGV’s safety and providing a relatively smoother driving experience. Therefore, the AEB algorithm undoubtedly offers greater advantages in complex environments, particularly in terms of preventing emergency stops.

## 4. Discussion

This paper proposes an improved AEB algorithm for AGVs, aimed at addressing issues such as mechanical wear, shortened battery life, navigation error accumulation, and reduced operational efficiency caused by frequent starts and stops in industrial environments. The algorithm adopts a multi-stage deceleration strategy, significantly reducing the number of emergency stops in dynamic environments, thereby improving the system’s operational smoothness. In addition to its applicability for straight-line driving, the AEB algorithm has been optimized for turning scenarios, automatically adjusting the deceleration detection range based on the turning trajectory to ensure smoothness and safety during turns. Furthermore, this paper presents a speed smoothing algorithm to prevent sudden velocity changes from impacting the system, ensuring a smooth transition during deceleration.

The experiments considered various operating conditions that challenge the AGV’s navigation and obstacle avoidance capabilities, including static object detection and acceleration/deceleration testing, turning obstacle avoidance testing, the testing of dynamic obstacle avoidance at intersections, a comparison of the TEB and AEB algorithms, and a comparison with traditional emergency stop strategies.

In the static object detection experiment, the AGV performed multi-stage deceleration under different acceleration and deceleration conditions, successfully reducing the number of emergency stops. By adjusting the acceleration and deceleration parameters, the experiment validated the AEB algorithm’s adaptability and performance advantages under various conditions. In the turning obstacle avoidance test, the AGV successfully implemented the AEB algorithm under different turning radii and speed conditions, avoiding collisions with obstacles and demonstrating the algorithm’s adaptability and robustness with complex paths. In the experiment on dynamic obstacle avoidance at intersections, the AGV avoided collisions and achieved a high emergency stop avoidance rate. In the comparison experiment of the TEB and AEB algorithms, by comparing their performance in a dynamic obstacle environment, it was demonstrated that AEB achieved better motion smoothness and efficiency without relying on path adjustment. In the comparison with traditional emergency stop strategies, the AEB algorithm reduced the emergency stop rate by an average of 42.526% throughout the experiment, highlighting its advantage in preventing emergency stops.

Despite the improvements introduced by the AEB algorithm, several technical challenges and research gaps remain. One key challenge is the precise tuning of the acceleration and deceleration parameters, which requires careful calibration based on AGV characteristics, load variations, and operational constraints. Current methods lack an adaptive mechanism for real-time parameter optimization, making it difficult to maintain optimal performance across different AGV models and working conditions.

In low-speed industrial settings, such as automated assembly lines and warehouse logistics, the algorithm effectively enhances smooth navigation, reduces mechanical stress, and extends battery life by minimizing emergency stops. However, its reliance on predefined deceleration profiles limits its adaptability to unexpected environmental changes, leading to a gap in dynamic response optimization. In high-speed environments like airport baggage handling and large-scale logistics centers, where AGVs operate at higher velocities and encounter dynamic obstacles, the gradual deceleration approach may cause response delays when facing sudden obstructions. This highlights a major technical challenge in balancing braking smoothness with rapid response, requiring research into hybrid braking strategies that integrate aggressive braking when necessary while maintaining overall motion stability.

Additionally, in multi-AGV coordination scenarios, the algorithm’s independent braking decisions can lead to traffic inefficiencies, bottlenecks, or even deadlocks in environments with complex obstacle distributions. The absence of a collaborative braking mechanism creates a gap in fleet-level optimization, as current approaches lack the ability to consider global traffic flow and AGV interactions in cluttered spaces. Addressing this requires further research into cooperative braking strategies and fleet management algorithms that enhance overall system efficiency while preventing conflicts in obstacle-dense workspaces.

Future research will enhance the AEB algorithm’s adaptability, efficiency, and coordination. First, an adaptive mechanism for the real-time tuning of acceleration and deceleration parameters will be developed using machine learning or model-predictive control to optimize performance across different AGVs and environments. Second, hybrid braking strategies will be explored to balance smooth navigation with rapid response, enabling dynamic switching between gradual and aggressive braking based on real-time conditions. Lastly, cooperative braking strategies will be designed to improve multi-AGV coordination, integrating global traffic flow management to reduce congestion and prevent deadlocks in obstacle-dense environments.

## Figures and Tables

**Table 1:** AGV parameters.

Name	Value
Three-dimensional dimensions (L × W × H) (m)	0.6 × 0.6 × 0.3
Weight (kg)	27
Minimum ground clearance (mm)	15
Maximum linear velocity (m/s)	1.8
Maximum acceleration (m/s^2^)	1
Maximum deceleration (m/s^2^)	1
Heading accuracy (°)	±1
Stop accuracy (mm)	±10
Repeatability accuracy (mm)	±10
Radar mounting height (m)	0.2
Radar scanning angle (°)	270
Radar scanning frequency (Hz)	10

**Table 2:** Baseline parameters.

Name	Value
Footprint (m)	[[0.3, 0.3], [0.3, −0.3], [−0.3, −0.3], [−0.3, 0.3]]
Emergency_stop_footprint (m)	[[0.4, 0.4], [0.4, −0.4], [−0.4, −0.4], [−0.4, 0.4]]
Dis_spacing (m)	0.1
Acceletation (m/s^2^)	0.3 m/s^2^
Deceleration (m/s^2^)	0.3 m/s^2^
Aeb_obstacle_distance (m)	[0.5, 1.0, 1.5, 2.0, 2.5]
Aeb_obstacle_speed (m)	[0.1, 0.3, 0.5, 0.7, 0.9]

**Table 3:** Comparison of acceleration and deceleration when acceleration changes.

Set Value (m/s^2^)		Actual Value (m/s^2^)						
a	d	a	$\bar{d}$	$d_{1}$	$d_{2}$	$d_{3}$	$d_{4}$	$d_{5}$
0.3	0.3	0.54	0.542	0.47	0.55	0.57	0.61	0.51
0.4	0.3	0.64	0.568	0.71	0.54	0.49	0.54	0.56
0.5	0.3	1.09	0.706	0.71	0.69	0.74	0.65	0.74
0.6	0.3	1.19	0.730	0.71	0.74	0.69	0.74	0.77
0.7	0.3	1.23	0.620	0.48	0.63	0.67	0.65	0.67
0.8	0.3	1.33	0.674	0.71	0.69	0.67	0.63	0.67

**Table 4:** Acceleration and deceleration values when deceleration changes.

Set Value (m/s^2^)		Actual Value (m/s^2^)						
a	d	a	$\bar{d}$	$d_{1}$	$d_{2}$	$d_{3}$	$d_{4}$	$d_{5}$
0.3	0.3	0.54	0.542	0.47	0.55	0.57	0.61	0.51
0.3	0.4	0.46	0.726	0.77	0.71	0.74	0.74	0.67
0.3	0.5	0.54	1.225	1.15	/	1.11	1.53	1.11
0.3	0.6	0.46	1.293	1.36	/	1.05	1.33	1.43
0.3	0.7	0.58	1.588	1.43	/	1.43	1.82	1.67
0.3	0.8	0.50	1.753	1.88	/	0.91	2.00	2.22

**Table 5:** The selected values for the parameters of the AGV in the experiment on dynamic obstacle avoidance at intersections.

Name	Value
Footprint (m)	[[0.3, 0.3], [0.3, −0.3], [−0.3, −0.3], [−0.3, 0.3]]
Emergency_stop_footprint (m)	[[0.4, 0.4], [0.4, −0.4], [−0.4, −0.4], [−0.4, 0.4]]
Dis_spacing (m)	0.1
Aeb_obstacle_distance (m)	[0.5, 1.0, 1.5, 2.0, 2.5]
Aeb_obstacle_speed (m)	[0.1, 0.3, 0.5, 0.7, 0.9]
Selected values of the velocity (m/s)	0.4
Selected values of the acceleration (m/s^2^)	0.4
Holding_time (s)	0.0

**Table 6:** The emergency stop avoidance rate under different conditions.

	Obstacle	2 to 3	2 to 4	3 to 4
AGV				
**1 to 2**		60%	100%	100%
**1 to 3**		80%	0%	100%
**1 to 4**		100%	40%	80%

**Table 7:** Key parameter settings for AEB and TEB.

Name	Value
Footprint (m)	[[0.3, 0.3], [0.3, −0.3], [−0.3, −0.3], [−0.3, 0.3]]
Emergency_stop_footprint (m)	[[0.4, 0.4], [0.4, −0.4], [−0.4, −0.4], [−0.4, 0.4]]
Dis_spacing (m)	0.1
Acceletation (m/s^2^)	0.3 m/s^2^
Deceleration (m/s^2^)	0.3 m/s^2^
Aeb_obstacle_distance (m)	[0.5, 1.0, 1.5, 2.0, 2.5]
Aeb_obstacle_speed (m)	[0.1, 0.3, 0.5, 0.7, 0.9]
Enable_homotopy_class_planning	False
Min_obstacle_dist (m)	0.5
Inflation_dist (m)	0.6
Weight_obstacle	0
Weight_dynamic_obstacle	10
Delete_detours_backwards	True
Max_vel_x (m/s)	0.3, 0.4, 0.5, 0.6, 0.7

**Table 8:** Travel time of different algorithms at various set speeds.

	Speed	0.3 m/s	0.4 m/s	0.5 m/s	0.6 m/s	0.7 m/s
Algorithm						
**TEB**		14.02	11.09	10.24	10.21	9.97
**AEB**		14.80	12.25	10.89	10.03	9.87

**Table 9:** The selected values for the parameters of the two obstacle avoidance strategies and the experimental variables.

Name	Value
Footprint (m)	[[0.3, 0.3], [0.3, −0.3], [−0.3, −0.3], [−0.3, 0.3]]
Emergency_stop_footprint (m)	[[0.4, 0.4], [0.4, −0.4], [−0.4, −0.4], [−0.4, 0.4]]
Dis_spacing (m)	0.1
Aeb_obstacle_distance (m)	[0.5, 1.0, 1.5, 2.0, 2.5]
Aeb_obstacle_speed (m)	[0.1, 0.3, 0.5, 0.7, 0.9]
Normal_obstacle_distance (m)	[0.5]
Normal_obstacle_speed (m)	[0.0]
Selected values of the obstacle distance (m)	3, 4, 5, 6, 7
Selected values of the velocity (m/s)	0.3, 0.4, 0.5, 0.6, 0.7
Selected values of the acceleration (m/s^2^)	0.3, 0.4, 0.5, 0.6, 0.7

**Table 10:** The average value of $\bar{R_{ESA}}$ corresponding to obstacles at different distances and the overall average value.

The Obstacle Distance (m)	$\bar{R_{ESA}}$
3	49.37%
4	43.77%
5	43.25%
6	40.64%
7	35.60%
Average value	42.526%

## Data Availability

Due to privacy restrictions, the data are not publicly available. However, access can be granted upon reasonable request.

## Abbreviations

The following abbreviations are used in this manuscript:

AGV	automated guided vehicle
AEB	Autonomous Emergency Braking
SLAM	Simultaneous Localization and Mapping
GPS	Global Positioning System
TTC	Time to Collision
ROS	Robot Operating System
TEB	Timed-Elastic-Band
ESA	emergency stop avoidance
